# Antibacterial Evaluation, In Silico Characters and Molecular Docking of Schiff Bases Derived from 5-aminopyrazoles

**DOI:** 10.3390/molecules24173130

**Published:** 2019-08-28

**Authors:** Ashraf S. Hassan, Ahmed A. Askar, Eman S. Nossier, Ahmed M. Naglah, Gaber O. Moustafa, Mohamed A. Al-Omar

**Affiliations:** 1 Organometallic and Organometalloid Chemistry Department, National Research Centre, Dokki, Cairo 12622, Egypt; 2 Botany and Microbiology Department, Faculty of Science (Boys), Al-Azhar University, Cairo 11751, Egypt; 3 Department of Pharmaceutical Chemistry, Faculty of Pharmacy (Girls), Al-Azhar University, Cairo 11754, Egypt; 4 Department of Pharmaceutical Chemistry, Drug Exploration and Development Chair (DEDC), College of Pharmacy, King Saud University, Riyadh 11451, Saudi Arabia; 5 Peptide Chemistry Department, National Research Centre, Dokki, Cairo 12622, Egypt

**Keywords:** Schiff bases, Antibacterial, 5-Aminopyrazole, *Staphylococcus aureus* DNA gyrase, Dihydrofolate reductase, Molecular docking

## Abstract

A series of Schiff bases **14**–**25** were designed and synthesized for evaluation of their antibacterial properties against multi-drug resistant bacteria (MDRB). The antibacterial activities of Schiff bases **14**–**25** showed that most of the synthesized compounds displayed a significant antibacterial activity. Assessment of in silico ADMET properties (absorption, distribution, metabolism, excretion and toxicity) of Schiff bases illustrates that all derivatives showed agreement to the Lipinski’s rule of five. Further enzymatic assay aided by molecular docking study demonstrated that compound **18** is a potent inhibitor of *staphylococcus aureus* DNA gyrase and dihydrofolate reductase kinases. This study could be valuable in the discovery of new potent antimicrobial agents.

## 1. Introduction

Antibiotics, anti-microbial drugs, and anti-infectious agents are used for treating infectious with micro-organism diseases and able to kill or inhibit the growth of microbes by inhibition of cell membranes synthesis, protein synthesis, nucleic acid synthesis, or cytoplasmic membranes. Recently, the resistance of microbes to antibiotics can be observed and classified into internal resistance and acquired resistance. Inactivation of drugs by bacterial enzymes or the drug cannot bind are the reasons which explained the biochemical mechanisms of internal and acquired resistances. Therefore, there is an urgent need for production of new antimicrobial drugs or develop the used drugs to oppose the mutation of the microbes to solve the resistance.

**Figure 1:**
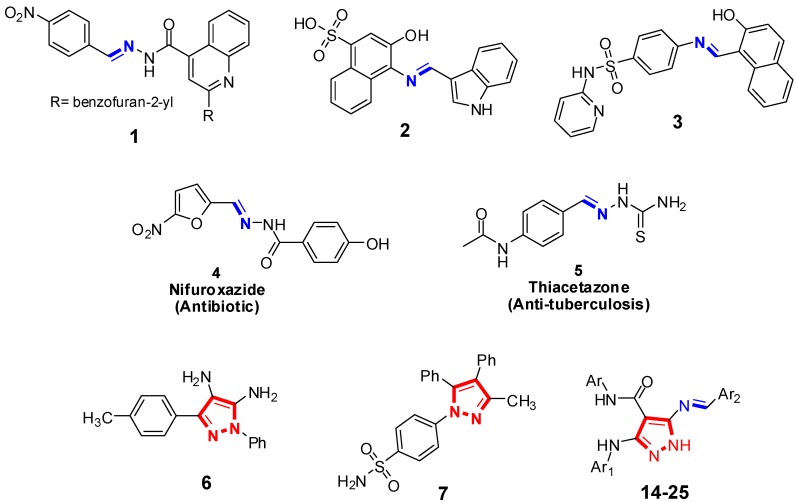
Structures of the antimicrobial Schiff bases **1**–**3**, Nifuroxazide **4**, Thiacetazone **5**, pyrazole derivatives **6, 7** and the target Schiff bases **14**–**25**.

Schiff bases (bearing imine or azomethine–C=N–) have shown a broad spectrum of activities including anti-diabetic, enzyme inhibition, DNA binding, cleavage activity and cytotoxicity activities [1,2,3,4,5,6]. Additionally, there are several reports that highlight the importance of Schiff bases as antimicrobial agents [7,8,9,10,11]. Compound **1** demonstrated significant antibacterial activity against *S. aureus* and *E. faecalis* [12]. Compound **2** showed good antimicrobial activity against *B. subtilis, P. fluorescence*, and *S. aureus* [13]. Also, compound **3** exhibited better antimicrobial activity against *S. aureus* and *S. pyogenes* [14]. In fact, the azomethine group is found on some marketed drugs e.g., Nifuroxazide (INN) **4** and Thiacetazone **5** are an oral antibiotic, which are used in the treatment of tuberculosis (Figure 1). 

Many pyrazole compounds are characterized by their biological activities [15,16,17,18], especially antimicrobial activities such as compounds **6** and **7** exhibit antimicrobial activities [19,20]. (Figure 1) 

From the above biological effectiveness of Schiff bases as well as our target to display the biological activities of compounds [21,22,23,24,25,26,27,28,29,30,31,32,33,34,35,36,37,38,39], we have reported in this work a series of Schiff bases **14**–**25** was synthesized by the reaction of 5-amino-pyrazoles **12a**–**c** with aldehydes **13a**–**d** (Figure 1) for evaluation of their antibacterial properties against multi-drug resistant bacteria (MDRB). In addition to this, enzymes assay (*staphylococcus aureus* DNA gyrase, topoisomerase IV and dihydrofolate reductase enzymes), the molecular modeling study and structure-activity relationship were carried out.

## 2. Results and Discussion 

### 2.1. Chemistry

**Scheme 1:**
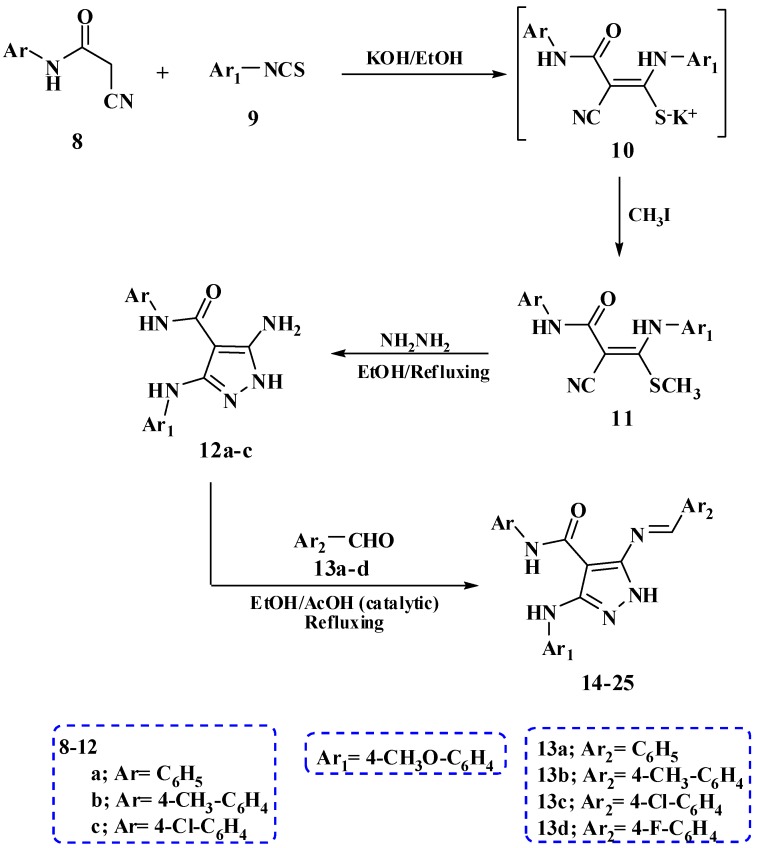
Synthesis of Schiff bases **14**–**25**.

5-Amino-1*H*-pyrazoles **12a**–**c** were prepared *via* the sequence reaction of *N*-substituted cyanoacetamides **8** with 4-methoxyisothicyanate (**9**), methyl iodide and then with hydrazine hydrate in ethanol refluxing. A series of Schiff bases **14**–**25** were synthesized by the condensation of 5-aminopyrazoles **12a**–**c** with aromatic aldehydes **13a**–**d** and the chemical structures wer confirmed *via* spectral data (Scheme 1 and Table 1).

### 2.2. Antibacterial Evaluation

In vitro antibacterial activities against multi-drug resistant bacteria (MDRB) of Schiff bases **14**–**25** were performed at botany and microbiology department, Faculty of Science, Al-Azhar University, Cairo, Egypt. The antibacterial potential of **14**–**25** were investigated towards the multi-drug resistant bacteria (MDRB). The results were summarized as the diameter of the inhibition zones in mm [40] and minimal inhibitory concentration (MIC, µg/mL) [41] values in Table 2.

**Figure 2:**
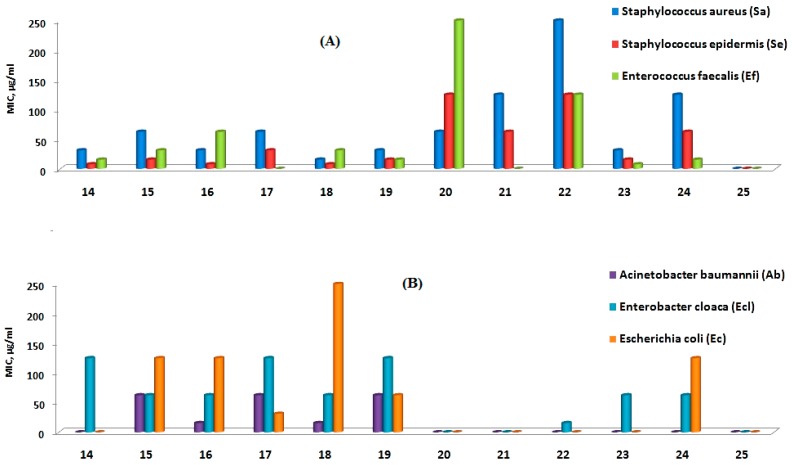
Minimal inhibitory concentrations (MIC, µg/mL) of Schiff bases **14**–**25** against multi-drug resistant bacteria (**A**) Gram-positive bacteria, (**B**) Gram-negative bacteria.

The result of the minimal inhibitory concentration (MIC) values was in Figure 2. We could see that Schiff base **18** showed very good activity against *Staphylococcus aureus* (MIC: 15.62 µg/mL), while compounds **14**, **16**, **19** and **23** (MIC: 31.25 µg/mL) showed good activity and Schiff bases **15**, **17** and **20** exhibited moderate activity with MIC = 62.5 µg/mL. Compounds **14**, **16** and **18** (MIC: 7.81 µg/mL) showed significant activity against *Staphylococcus epidermis* (Sp) while compounds **15, 19**, and **23** showed very good activity (MIC: 15.62 µg/mL). Schiff base **17** (MIC: 31.25 µg/mL) showed good activity.

In the case of *Enterococcus faecalis* (Ef), Schiff bases **23** (MIC: 7.81 µg/mL) showed significant activity and Schiff bases **14**, **19** and **24** very good activity (MIC: 15.62 µg/mL), while compounds **15** and **18** (MIC: 31.25 µg/mL) showed good activity. Schiff base **16** (MIC: 62.5 µg/mL) showed moderate activity.

In the case of *Acinetobacter baumannii* (Ab), Schiff bases **16** and **18** showed very good activity (MIC: 15.62 µg/mL), while compounds **15**, **17**, and **19** (MIC: 62.5 µg/mL) showed moderate activity.

Schiff base **22** displayed very good activity (MIC: 15.62 µg/mL) against *Enterobacter cloaca* (Ecl), while compounds **15**, **16**, **18**, **23** and **24** (MIC: 62.5 µg/mL) showed moderate activity.

Schiff base **17** (MIC: 31.25 µg/mL) showed good activity, while **19** (MIC: 62.5 µg/mL) showed moderate activity against *Escherichia coli* (Ec).

### 2.3. Structure-Activity Relationship (SAR)

**Figure 3:**
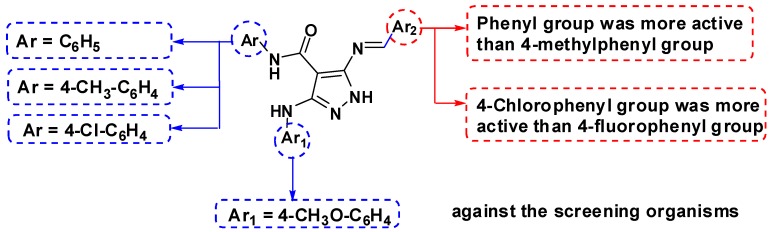
A brief Structure activity relationship (SAR) study of Schiff bases **14**–**25**.

From the results of antibacterial activities of Schiff bases **14**–**25** against multi-drug resistant bacteria, it was found that, in case of Ar = Ph, 4-CH_3_-C_6_H_4_ or 4-Cl-C_6_H_4_, the order of antibacterial activity Ar_2_ = Ph > 4-CH_3_-C_6_H_4_ and Ar_2_ = 4-Cl-C_6_H_4_ > 4-F-C_6_H_4_ was observed upon screening of Schiff bases **14**–**25** against the screening organisms (Figure 3).

### 2.4. In Silico ADMET Properties of Schiff Bases 14-25

The physical properties and the ADMET parameters (absorption, distribution, metabolism, excretion and toxicity) of Schiff bases **14**–**25** were computed using the freely accessible web server Swiss ADME (http://swissadme.ch/index.php#undefined). The results of in silico ADMET properties of Schiff bases **14**–**25** are listed in Table 3. 

The molecular weight (MW), the number of hydrogen bond acceptors (nHBA), donors (nHBD), the number of rotatable bonds (nRB) and the topological polar surface area (TPSA) for all the Schiff bases were in accordance with the Lipinski’s rule of five. The lipophilicity property (expressed as MLogP ≤ 4.15) was in the range for all the Schiff bases excluding **20**, **21**, **23**, **24** and **25**. The highly lipophilic character (MLogP > 4.15) of the compounds **20**, **21**, **23**, **24** and **25** may be because of the presence of chloro or fluoro atoms in their structures which may make difficult their transport through the blood strain [42].

### 2.5. In Vitro Kinase Assessment 

In an effort to study the preliminary mechanism of the compound **18** with potent antibacterial activity, an enzyme inhibitory assay was performed towards *staphylococcus aureus* DNA gyrase, topoisomerase IV and dihydrofolate reductase enzymes. The obtained results were presented as IC_50_ and provided in Table 4 using suitable positive controls, Ciprofloxacin and Methotrexate.

From Table 4, it was observed that compound **18** demonstrated a nearly equipotent inhibitory activity towards DNA gyrase and weak activity against topoisomerase IV in comparison with the reference Ciprofloxacin (IC_50_ = 1.68 ± 0.10, 74.55 ± 1.20, 1.51 ± 0.18 and 24.14 ± 1.01 μM, respectively). Moreover, compound **18** revealed two folds increase in the suppression effect towards dihydrofolate reductase comparing with Methotrexate (IC_50_ = 0.08 ± 1.15 and 0.14 ± 1.07 μM, respectively).

### 2.6. Molecular Docking Study

**Figure 4:**
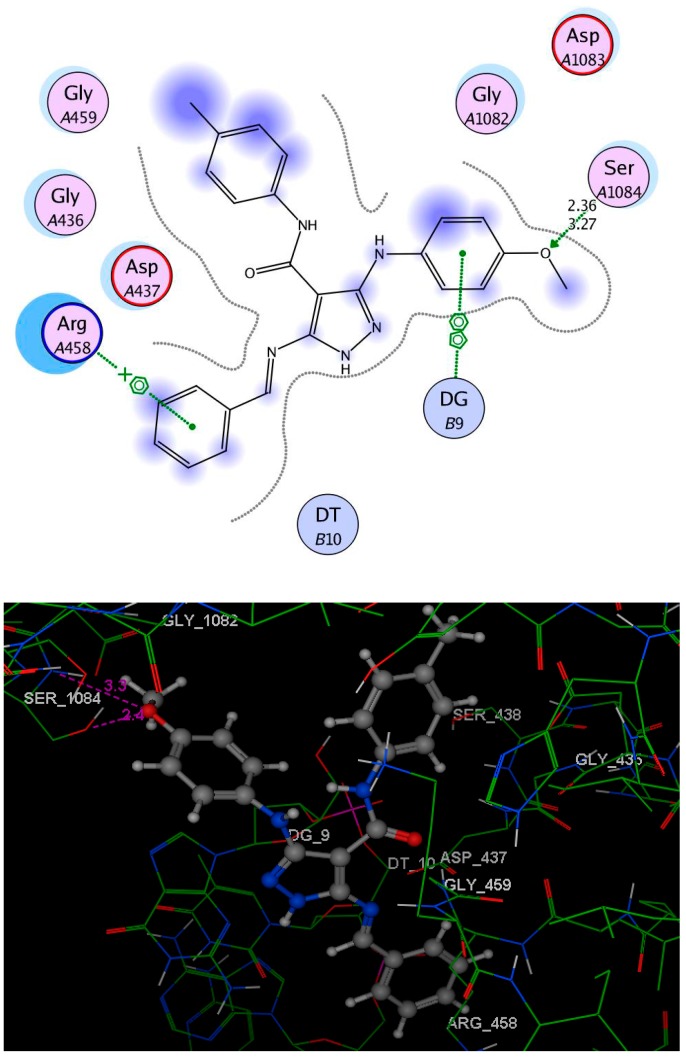
2D and 3D interaction diagrams of compound **18** with *Staphylococcus aureus* DNA gyrase (PDB code: 2XCT) (hydrogen bonds are illustrated as arrows, C atoms are colored gray, N blue, and O red).

**Figure 5:**
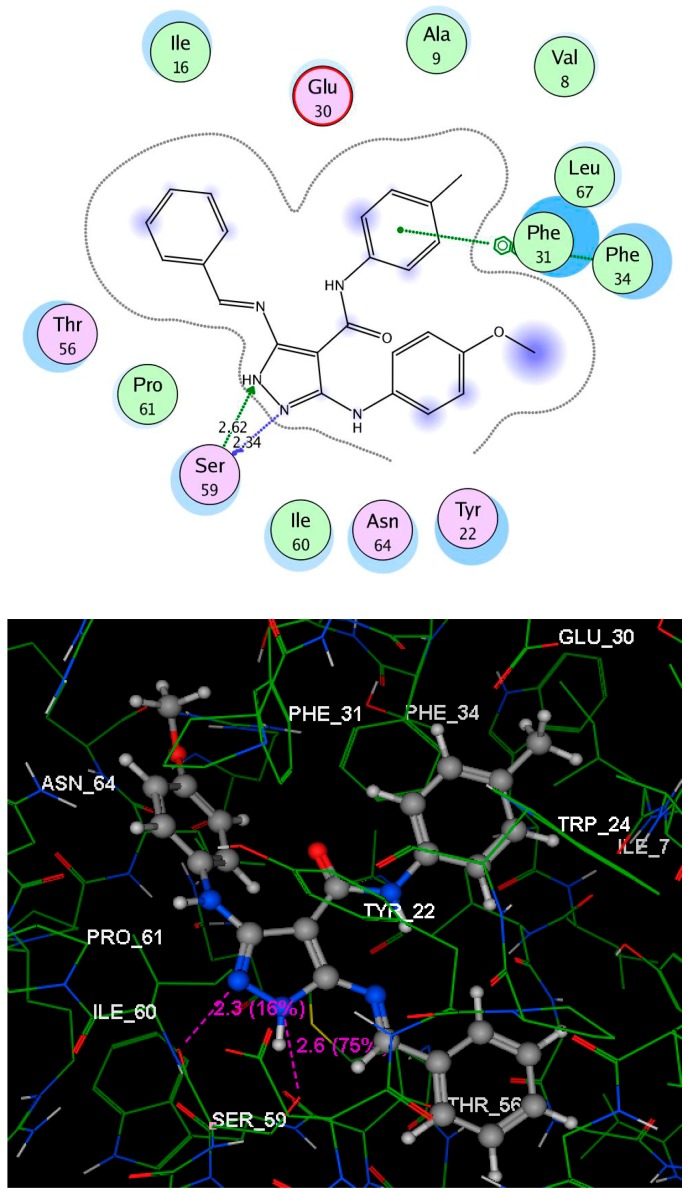
2D and 3D interaction diagrams of compound **18** with DHFR (PDB code: 1DLS) (hydrogen bonds are illustrated as arrows, C atoms are colored gray, N blue, and O red).

Molecular docking studies concerning the in vitro kinase assessment were performed to understand the interactions of compound **18** with *Staphylococcus aureus* DNA gyrase and Dihydrofolate reductase enzymes. The binding modes of compounds **18** were investigated through using Molecular Operating Environment (MOE^®^) 2008.10 [43]. The X-ray crystal structures of *Staphylococcus aureus* DNA gyrase (PDB code: 2XCT) [44] and dihydrofolate reductase (PDB code: 1DLS) [45] were downloaded from the Protein Data Bank. In the present study, the proposed docking algorithms were initially validated by self-docking of the co-crystallized ligands Ciprofloxacin and Methotrexate to each of the aforementioned targets and exhibited root mean square deviation (RMSD) values of 0.86 and 0.92 A˚, respectively. Subsequently, docking procedures have been achieved for compound **18** and the corresponding 2D and 3D representations of the binding modes are illustrated in Figure 4 and Figure 5.

As shown in Figure 4, compound **18** linked tightly with amino acid residues of *Staphylococcus aureus* DNA gyrase. There were arene-arene interactions established between the centroids of DG9 and 4-methoxyphenyl, and arene-cation interactions between the centroid of benzylidene at p-5 of pyrazole moiety and Arg458. Moreover, the oxygen of 4-methoxy group supported the binding with two hydrogen bonds with the sidechain of Ser1084 (distance: 2.36 and 3.27 Å). 

Considering the binding interaction of compound **18** with dihydrofolate reductase illustrated in Figure 5, it was noticed that N1 and N2 of pyrazole scaffold participated by two hydrogen bonds with the sidechain and the backbone of Ser59 (distance: 2.62 and 2.34 Å, respectively). Additionally, the tolyl moiety formed arene-arene interaction with the centroid of Ph34 passing through Ph31.

## 3. Materials and Methods

### 3.1. Chemicals

5-Amino-1*H*-pyrazoles **12a**–**c** [46] and Schiff bases **14**–**25** [47] were prepared according to the reported procedure. 

The chemical structures of Schiff bases **14**–**25** was confirmed *via* spectral data [47].

### 3.2. In Vitro Antibacterial Evaluation 

#### 3.2.1. Test Microorganisms

The synthesized compounds, Schiff bases **14**–**25**, were in vitro evaluation of their antibacterial properties against multi-drug resistant bacteria (MDRB). Examples of Gram-positive bacteria are *Staphylococcus aureus* (MRSA, Sa), *Staphylococcus epidermis* (Sp), and *Enterococcus faecalis* (Ef). Examples of Gram-negative bacteria are *Acinetobacter baumannii* (Ab), *Enterobacter cloaca* (Ecl), and *Escherichia coli* (Ec). All the tested strain was identified by Vitek^®^2 system. The multi-drug resistant to antibiotics such as Ampicillin, Cephalexin, Colisin, Ipemenem, and Meropenem was verified. 

#### 3.2.2. Antibacterial Activity

In vitro antibacterial activities were performed at botany and microbiology department, Faculty of Science, Al-Azhar University, Cairo, Egypt. The antibacterial potential of Schiff bases **14**–**25** were investigated towards multi-drug resistant bacteria (MDRB) and expressed as the diameter of the inhibition zones according to the agar plate diffusion method [40]. 

#### 3.2.3. Minimum Inhibitory Concentration (MIC) of the Active Compounds

The minimal inhibitory concentration (MIC) of the most potent Schiff bases was determined by the conventional paper disk diffusion method [41].

### 3.3. In Vitro Kinase Assessment 

The in vitro enzyme inhibition determination for compound **18** was carried out in the confirmatory diagnostic unit, Vacsera, Egypt. The evaluation performed profiling of compound **18** against *Staphylococcus aureus* DNA gyrase, topoisomerase IV, and dihydrofolate reductase enzymes using Ciprofloxacin and Methotrexate as reference drugs according to the previously reported method [45,48].

### 3.4. Molecular Docking Study

Automated docking studies were carried out using Molecular Operating Environment (MOE^®^) 2008.10 [43]. The crystal structures of *Staphylococcus aureus* DNA gyrase (PDB code: 2XCT) [44] and dihydrofolate reductase (PDB code: 1DLS) [45] complexed with Ciprofloxacin and Methotrexate, respectively were retrieved from the RCSB Protein Data Bank (http://www.rcsb.org/pdb/home/home.do). 

## 4. Conclusions

In this work, a series of Schiff bases **14**–**25** were synthesized by the condensation of 5-aminopyrazoles **12a**–**c** with aromatic aldehydes **13a**–**d**, with high yields for evaluation of their in vitro antibacterial activities against multi-drug resistant bacteria (MDRB). In general, most of Schiff bases **14**–**25** displayed better antibacterial activity. In addition, a positive result of kinase inhibition was implicated by molecular docking study against *Staphylococcus aureus* DNA gyrase and dihydrofolate reductase enzymes. Furthermore, drug-likeness data revealed that the studied compounds fulfill Lipinski’s rule requirements and have good drug score values. These preliminary results of Schiff bases against multi-drug resistant bacteria (MDRB) could provide an exceptional model that may lead to the discovery of new antibiotics by derivatization or modification.

## Figures and Tables

**Table 1:** Schiff bases **14**–**25**.

Compounds	Ar	Ar_1_	Ar_2_
**14**	Ph	4-MeOC_6_H_4_-	Ph
**15**	Ph	4-MeOC_6_H_4_-	4-MeC_6_H_4_-
**16**	Ph	4-MeOC_6_H_4_-	4-ClC_6_H_4_-
**17**	Ph	4-MeOC_6_H_4_-	4-FC_6_H_4_-
**18**	4-MeC_6_H_4-_	4-MeOC_6_H_4_-	Ph
**19**	4-MeC_6_H_4_-	4-MeOC_6_H_4_-	4-MeC_6_H_4_-
**20**	4-MeC_6_H_4_-	4-MeOC_6_H_4_-	4-ClC_6_H_4_-
**21**	4-MeC_6_H_4_-	4-MeOC_6_H_4_-	4-FC_6_H_4_-
**22**	4-ClC_6_H_4_-	4-MeOC_6_H_4_-	Ph
**23**	4-ClC_6_H_4_-	4-MeOC_6_H_4_-	4-MeC_6_H_4_-
**24**	4-ClC_6_H_4_-	4-MeOC_6_H_4_-	4-ClC_6_H_4_-
**25**	4-ClC_6_H_4_-	4-MeOC_6_H_4_-	4-FC_6_H_4_-

**Table 2:**
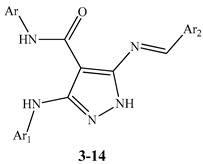
Minimal inhibitory concentrations in µg/mL of Schiff bases against multi-drug resistant bacteria (MDRB).

Comp.	Ar	Ar_1_	Ar_2_	Gram-Positive Bacteria			Gram-Negative Bacteria		
				Sa	Se	Ef	Ab	Ecl	Ec
**14**	Ph	4-MeOC_6_H_4_-	Ph	31.25	7.81 *	15.62	NA	125	NA
**15**	Ph	4-MeOC_6_H_4_-	4-MeC_6_H_4_-	62.50	15.62	31.25	62.5	62.5	125
**16**	Ph	4-MeOC_6_H_4_-	4-ClC_6_H_4_-	31.25	7.81 *	62.5	15.62 *	62.5	125
**17**	Ph	4-MeOC_6_H_4_-	4-FC_6_H_4_-	62.5	31.25	NA	62.5	125	31.25 *
**18**	4-MeC_6_H_4_-	4-MeOC_6_H_4_-	Ph	15.62 *	7.81 *	31.25	15.62 *	62.5	250
**19**	4-MeC_6_H_4_-	4-MeOC_6_H_4_-	4-MeC_6_H_4_-	31.25	15.62	15.62	62.5	125	62.5
**20**	4-MeC_6_H_4_-	4-MeOC_6_H_4_-	4-ClC_6_H_4_-	62.5	125	250	NA	NA	NA
**21**	4-MeC_6_H_4_-	4-MeOC_6_H_4_-	4-FC_6_H_4_-	125	62.5	NA	NA	NA	NA
**22**	4-ClC_6_H_4_-	4-MeOC_6_H_4_-	Ph	250	125	125	NA	15.62 *	NA
**23**	4-ClC_6_H_4_-	4-MeOC_6_H_4_-	4-MeC_6_H_4_-	31.25	15.62	7.81 *	NA	62.5	NA
**24**	4-ClC_6_H_4_-	4-MeOC_6_H_4_-	4-ClC_6_H_4_-	125	62.5	15.62	NA	62.5	125
**25**	4-ClC_6_H_4_-	4-MeOC_6_H_4_-	4-FC_6_H_4_-	NA	NA	NA	NA	NA	NA
**Ciprofloxacin**				7.81 *	15.62 *	7.81 *	15.62 *	15.62 *	7.81 *

Comp.: Compound. Gram-positive bacteria: *Staphylococcus aureus* (MRSA, Sa); *Staphylococcus epidermis* (Se) and *Enterococcus faecalis* (Ef). Gram-negative bacteria: *Acinetobacter baumannii* (Ab); *Enterobacter cloaca* (Ecl) and *Escherichia coli* (Ec). NA: No Activity. * The most potent compound compared to others.

**Table 3:** In silico prediction of Lipinski‘s rule of five for the Schiff bases **14**–**25**.

Comp.	MW ^a^	*M*LogP ^b^	*n*HBA ^c^	nHBD ^d^	*n*RB ^e^	TPSA ^f^	n*_violations_* ^g^
Rule	<500	≤4.15	≤10	≤5	≤10	<160 Å^2^	0
**14**	411.46	3.65	4	3	8	91.40	0
**15**	425.48	3.86	4	3	8	91.40	0
**16**	445.90	4.13	4	3	8	91.40	0
**17**	429.45	4.02	5	3	8	91.40	0
**18**	425.48	3.86	4	3	8	91.40	0
**19**	439.51	4.06	4	3	8	91.40	0
**20**	459.93	*4.33*	4	3	8	91.40	1
**21**	443.47	4.23	5	3	8	91.40	1
**22**	445.90	4.13	*4*	3	8	91.40	0
**23**	459.93	4.33	4	3	8	91.40	1
**24**	480.35	4.60	4	3	8	91.40	1
**25**	463.89	4.50	5	3	8	91.40	1

^a^ Molecular Weight; ^b^ Calculated Lipophillicity (MLog *P*_o/w_); ^c^ Number of Hydrogen Bond Acceptor; ^d^ Number of Hydrogen Bond Donor; ^e^ Number of Rotatable Bond; ^f^ Topological Polar Surface Area; ^g^ Violations from Lipinski’s Rule.

**Table 4:** Inhibitory assessment (IC_50_ in μM) of compound **18** on *Staphylococcus aureus* DNA gyrase, Topoisomerase IV and Dihydrofolate reductase enzymes.

Compound	IC_50_ (Mean ± SEM) (µM)		
	DNA Gyrase	Topoisomerase IV	DHFR
**18**	1.68 ± 0.10	74.55 ± 1.20	0.08 ± 1.15
**Ciprofloxacin**	1.51 ± 0.18	24.14 ± 1.01	-----
**Methotrexate**	-----	-----	0.14 ± 1.07

IC_50_: Compound concentration required to inhibit the enzyme viability by 50%, SEM = standard error mean; each value is the mean of three values.
